# Early career psychiatrists’ ability to evaluate and manage negative symptoms of schizophrenia – Results from a European survey

**DOI:** 10.1192/j.eurpsy.2025.10142

**Published:** 2025-12-17

**Authors:** Anna Julia Krupa, Marcin Siwek, Tomasz Gondek, Gaia Sampogna, Renato de Filippis, Aistė Lengvenytė, Beren Özel, Cristiana Tăpoi, Visnja Banjac Baljak, Stefan Jerotic, Ruta Karaliuniene, Jesper Nørgaard Kjær, Giulio Longo, Petra Marinova-Djambazova, Sara Medved, Miloš Milutinović, Inés Oliveira, Dorottya Őri, Mariana Pinto da Costa, Diego Quattrone, Jördis Rausch, Georgios Schoretsanitis, Ekin Sönmez Güngör, Asilay Seker, Zbigniew Sołtys, Anna Szczegielniak, Jelena Vrublevska, Michael Wallies, Dominika Dudek, Andrea Fagiolini, Armida Mucci, Silvana Galderisi

**Affiliations:** 1 Department of Biological and Community Psychiatry, Jagiellonian University Medical College Krakow, Poland; 2 Institute of Social Studies, University of Lower Silesia, Wroclaw, Poland; 3 University of Campania Luigi Vanvitelli, Naples, Italy; 4 Psychiatry Unit, Department of Health Sciences, Magna Graecia University of Catanzaro, Catanzaro, Italy; 5 Department of Emergency Psychiatry and Acute Care, Lapeyronie Hospital, CHU Montpellier, Montpellier, France; 6 Institute of Functional Genomics, University of Montpellier, CNRS, INSERM, Montpellier, France; 7 Department of Psychiatry, Mus State Hospital, Türkiye; 8 Department of General Psychiatry, Alexandru Obregia Clinical Psychiatry Hospital, Bucharest, Romania; 9 Clinic of Psychiatry, University Clinical Center of the Republic of Srpska, Banjaluka, Bosnia and Herzegovina; 10 Faculty of Medicine, University of Banjaluka, Banjaluka, Bosnia and Herzegovina; 11 Faculty of Medicine, University of Belgrade, Belgrade, Serbia; 12 Rheinmosel Fachklinik, Landeskrankenhaus, Academic Hospital at Mainz University, Germany; 13 Department of Clinical Medicine, Aarhus University, Aarhus, Denmark; 14 Psychosis Research Unit, Aarhus University Hospital, Aarhus, Denmark; 15 Unit of Clinical Psychiatry, Department of Clinical Neurosciences/DIMSC, Polytechnic University of Marche, Ancona, Italy; 16 Department of Psychiatry, Medical University of Sofia, Sofia, Bulgaria; 17 Department of Psychiatry and Psychological Medicine, University Hospital Centre Zagreb: Zagreb, Croatia; 18 University Clinic of Psychiatry, Skopje, North Macedonia; 19 Hospital General Universitario Gregorio Marañón, Madrid, Spain; 20 Institute of Behavioural Sciences, Semmelweis University, Budapest, Hungary; 21 Department of Mental Health, Heim Pal National Pediatric Institute, Budapest, Hungary; 22 Institute of Psychiatry, Psychology & Neuroscience, King’s College London, London, United Kingdom; 23 Institute of Biomedical Sciences Abel Salazar, University of Porto, Porto, Portugal; 24 Social, Genetic and Developmental Psychiatry Centre, King’s College London Institute of Psychiatry Psychology & Neuroscience, London, United Kingdom; 25 Department of Mental Illnesses, Clinic of Psychiatry and Psychotherapy, Freiburg, Germany; 26 Department of Psychiatry, Psychotherapy and Psychosomatics, Hospital of Psychiatry, University of Zurich, Zurich, Switzerland; 27 The Zucker Hillside Hospital, Psychiatry Research, Northwell Health, Glen Oaks, NY, USA; 28 Zucker School of Medicine at Hofstra/Northwell, Hempstead, NY, USA; 29 Erenköy Mental Health and Neurological Diseases Training and Research Hospital, Department, Istanbul, Turkey; 30 Institute of Zoology and Biomedical Research, Laboratory of Experimental Neuropathology, Jagiellonian University, Krakow, Poland; 31 Department of Psychoprophylaxis, Faculty of Medical Sciences in ZabrzeMedical University of Silesia, Katowice, Poland; 32 Department of Neuromedicine and Neurosciences, Faculty of Medicine and Life Sciences, University of Latvia, Riga, Latvia; 33 Scientific Institute of Mental Health, National Centre of Mental Health, Riga, Latvia; 34 Therapie auf Augenhohe, Zurich, Switzerland; 35 Department of Adult Psychiatry, Jagiellonian University Medical College, Krakow, Poland; 36 Department of Molecular and Developmental Medicine, Division of Psychiatry, University of Siena, Siena, Italy

**Keywords:** (3–5) schizophrenia, clinical competence, clinical training in psychiatry, early career psychiatrist, negative symptoms

## Abstract

**Background:**

Negative symptoms (NS) represent an important unmet need in schizophrenia (SZ) assessment and management. Despite NS are strongly associated with poorer functioning and quality of life, they are frequently underrecognized, inconsistently evaluated, and show limited response to current treatments. Although specific assessment tools and European Psychiatric Association (EPA) guidance on NS have been developed, their impact on routine clinical practice appears limited. This study aimed to investigate the competence and confidence of European Early Career Psychiatrists (ECPs) in NS evaluation and management.

**Methods:**

The CARE project was a cross-sectional online survey directed towards ECPs from European countries.

**Results:**

828 ECPs’ responses were collected from 19 countries. The majority of ECPs were trainees (65.8%), reported theoretical training in negative symptoms (NS) and placements in schizophrenia-specialized settings (67.9% and 70.3%), while about half reported extracurricular NS training (51.1%) and involvement in clinical research (46.1%). Only 11% correctly identified NS domains, despite 65.7% felt well-trained in NS assessment tools. Just 15.9% correctly answered questions based on the EPA guidance papers. 46.7% and 25.9% ECPs reported feeling competent in NS evaluation and management, respectively. Gender (men) specialist status, research involvement, theoretical NS training, and placements in specialized SZ services predicted perceived competence. However, in-depth NS knowledge was predicted only by specialist status, engagement in clinical research, and extracurricular NS training.

**Conclusions:**

Despite reported exposure to NS training, ECPs demonstrated limited knowledge of NS. Actions need to be taken to ensure that ECPs receive the highest standard of training in NS.

## Introduction

Schizophrenia (SZ) is a serious mental illness affecting approximately 1% of the global population. SZ prevalence is over 10 times lower than that of depressive or anxiety disorders, and yet SZ ranks right next to depression and anxiety among the top three mental disorders contributing to disability worldwide [1]. Hence, there is a dire need to identify the barriers to the improvement of care and outcomes for individuals with SZ [2].

It should be noted that SZ might be multidimensional, including several subtypes with different neurobiological underpinnings, hence precise symptomatic assessment is crucial to develop its understanding [3, 4]. Negative symptoms (NS) were regarded as a core aspect of schizophrenia (SZ) when Bleuler and Kraepelin published the first comprehensive reports on this disorder in the 19th and 20th centuries. However, their presence was never considered mandatory for the diagnosis of SZ in standardized diagnostic systems (Diagnostic and Statistical Manual of Mental Disorders, International Classification of Diseases), and their role in the disorder assessment and treatment was marginalized [5]. A survey among European and American psychiatrists published in 2007 explored physicians’ perspectives on positive and negative symptoms of SZ and factors considered upon choosing antipsychotic treatment. Psychiatrists reported that efficacy for positive symptoms (90%) was the main driver of antipsychotic choice, while NS (62%) were less commonly mentioned. At the same time, the percentage of their SZ patients with inadequate control of particular symptoms reached as much as 71–77% for NS, and a lower number of 47–60% for positive symptoms [6]. Nevertheless, due to the longstanding efforts of dedicated researchers, the knowledge on NS neurobiology and its relevance to patient functioning has progressed [5, 7]. At the 2006 consensus conference of experts, a clear definition of NS was crafted and published. NS were defined as loss of normal functions and underscored as a distinct and often unmet treatment need [7], and the experts identified and defined the main NS domains, i.e., blunted affect, alogia, asociality, anhedonia, and avolition [6]. The consensus statement also highlighted the need for new, adequate tools to evaluate NS, given that the previous ones were no longer applicable to the current NS conceptualization [7]. Consequently, two rating scales were developed: the Brief Negative Symptoms Scale (BNSS) [8], and the Clinical Assessment Interview for Negative Symptoms (CAINS) [9]. The BNSS was later translated and validated into native languages of multiple European countries (e.g., Denmark, France, Germany, Italy, Netherlands, Norway, Poland, Portugal, Spain, Turkey) [10–20]. Moreover, the Schizophrenia Section of the European Psychiatric Association (EPA) published two guidance papers [21, 22] to support clinicians in the appropriate evaluation and management of NS. Later, the World Federation of Societies of Biological Psychiatry (WFSBP) and Canadian Network for Mood and Anxiety Treatments (CANMAT) Taskforce prepared the guidelines for clinicians on the use of nutraceuticals and phytoceuticals for psychiatric disorders, delineating precise data on the management of the negative symptoms of schizophrenia [23].

It remains unclear, however, if these efforts have been translated into teaching and everyday clinical practice. Hence, the CARE project was undertaken to explore the degree of knowledge and skills of early career psychiatrists (ECPs) in NS evaluation and management [24]. The pilot analysis of the CARE project, performed in the Polish ECPs sample, revealed poor knowledge and competence in the evaluation and management of NS, along with an expressed need for more training in NS [24]. This study aimed to investigate the ECPs’ competence and confidence in NS evaluation and management across different European Countries.

## Methods

Detailed information on the study methodology is reported in the published protocol of the CARE project [24]. Hereafter, we summarize the main aspects of the study design and methods. Throughout this article, the term ECPs will refer to psychiatry trainees, specialists within five years of completing their training and specialists under the age of 40.

### Study design

The CARE project is a cross-sectional online survey. The study was approved by the Research Ethics Committee of the Jagiellonian University Medical College in Krakow, Poland (No. of approval 118.0043.293.2024). The survey consisted of 23 questions, which covered ECPs’ demographic data, information about their training in NS, knowledge on NS, ability to use clinical tools to evaluate NS, and frequency of their use, familiarity with EPA and WFSBP/CANMAT guidance papers, sense of competence, and attitude towards assessing patients with various diagnoses, including SZ and SZ with persistent NS [24].

### Study group

Participants were eligible if they fulfilled the following criteria: 1) compliance with the definition of ECP (being a trainee psychiatrist under 40 years of age or a junior psychiatrist, i.e., less than 5 years after completing their specialty training), 2) practicing in a World Health Organization (WHO) European region country [25].

### Data collection

The online cross-sectional survey was distributed between January and June 2025, with the support of the national coordinators of EPA’s affiliated societies, National Psychiatric Associations (NPA), and the European Federation of Psychiatric Trainees (EFPT). The dissemination was facilitated via websites, mailing lists, social media, and other online platforms for psychiatrists, as well as via announcements at professional meetings/conferences for psychiatrists. A total of 869 individual responses were collected.

### Statistical analysis

The statistical analyses were performed using IBM SPSS Statistics (version 29.0.2.0 (20), 2024) and R software (version 4.4.3, 2025) [26, 27]. Initially, contingency tables and Chi-squared tests were employed to analyze the qualitative data. Given that the data did not present a normal distribution, the Mann Whitney-U tests were used to analyze the quantitative data.

The obtained responses were included in the analysis if the preplanned sample size of at least 25 or 15 independent observations for countries with populations>15 million or < 15 million citizens, respectively, was collected. The total number of included responses was 828. Responses from 7 of the 10 most populated European countries (Germany, the United Kingdom of Great Britain and Northern Ireland, France, Italy, Spain, Poland, Romania) and 12 less populated European countries were included (Table 1). The excluded observations (*n* = 41) were compared to the included ones to detect any significant difference (Supplementary Tables 1 and 2) [24].

**Table 1.** Demographic and training characteristics of participants

Demographic and training/work experience characteristics	Observations included in the analysis *n* = 828
Gender (women) *n* (%)	523 (63.2%)
Number of years of experience in the mental health system [median (IQR)]	4 (2–6)
Trainee status (vs. specialist) in adult or child and adolescent psychiatry *n* (%)	545 (65.8%)
Engagement in clinical research *n* (%)	382 (46.1%)
Theoretical courses on the NS of SZ were included in the specialist training curriculum *n* (%)	562 (67.9%)
Placements in clinics/wards specialized in SZ care and management were included in the specialist training curriculum *n* (%)	582 (70.3%)
Participated in additional theoretical or practical training in the NS of SZ assessment and management (outside of specialist training program) *n* (%)	423 (51.1%)
Country *n* (%)	Bosnia and Herzegovina 41 (5%) Bulgaria 19 (2.3%) Croatia 28 (3.4%) Denmark 39 (4.7%) France 35 (4.2%) Germany 40 (4.8%) Hungary 23 (2.8%) Italy 114 (13.8%) Latvia 19 (2.3%) Lithuania 16 (1.9%) North Macedonia 17 (2.1%) Poland 147 (17.8%) Portugal 15 (1.8%) Romania 61 (7.4%) Serbia 16 (1.9%) Spain 56 (6.8%) Switzerland 49 (5.9%) Turkey 64 (7.7%) United Kingdom of Great Britain and Northern Ireland 29 (3.5%)

Abbreviations: IQR, interquartile range; NS, negative symptoms; SZ, schizophrenia, US, United States of America.

The respondents excluded from the analysis were more specialists with longer experience in the mental health system, and a higher level of self-reported competence in managing NS. No other significant difference in demographics or main study variables linked to NS was identified between the included and excluded observations (Supplementary Tables 1 and 2).

Generalized linear models (GLMs) with binomial distribution and logit function were used to explore the predictors of dependent variables: 1) knowledge (defined as the total of correct responses to questions about NS domains identification and number of correct answers to the knowledge questions based on EPA and WFSBP/CANMAT guidance papers; range of correct responses = 0–5); 2) sense of competence (defined as the total number of responses to questions concerning evaluation and management of NS; range of responses 0–10). The independent variables for these GMLs included demographic characteristics (gender, trainee vs. specialist status, engagement in clinical research), characteristics of the specialist training (i.e., theoretical courses on NS in the curriculum, placements in clinics/wards specialized in SZ care in the curriculum, participation in extracurricular training in NS). Due to the low number of participants declaring a non-binary gender (*n* = 1) or preferring not to declare it (*n* = 5), these observations were excluded from the GLM analysis as they would not be statistically representative. In the models, the categories of reference for the independent variables were set as follows: gender – men status – specialist, engagement in clinical research – yes, theoretical courses on NS in specialist training curriculum – yes, placement in clinics/wards specialized in SZ care and management – yes, participation in extracurricular training in NS – yes. The data on gross domestic product (GDP) per capita were included in the analyses (among the independent variables) to verify whether the economic status of the ECPs’ country was significant to the ECPs’ knowledge and sense of competence in NS evaluation and management. The data about GDP per capita was based on the International Monetary Fund database [28].

## Results

### Sample characteristics

The general characteristics of the included sample are presented in Table 1. The sample included 828 responses from 19 European countries. The majority of the ECPs were women (63.2%), trainees (65.8%), with a median experience of 4 years in the mental health system. Nearly half (46.1%) of ECPs confirmed their engagement in clinical research. Most of them, 67.9 and 70.3%, respectively, confirmed that theoretical courses on NS and placements in clinics/wards specialized in SZ care and management were part of their specialist training curriculum. Furthermore, 51.1% reported having participated in extracurricular training in NS (Table 1).

Knowledge, skills, self-reported sense of competence in NS evaluation and management, and implementation of NS guidance.

**Figure 1.**
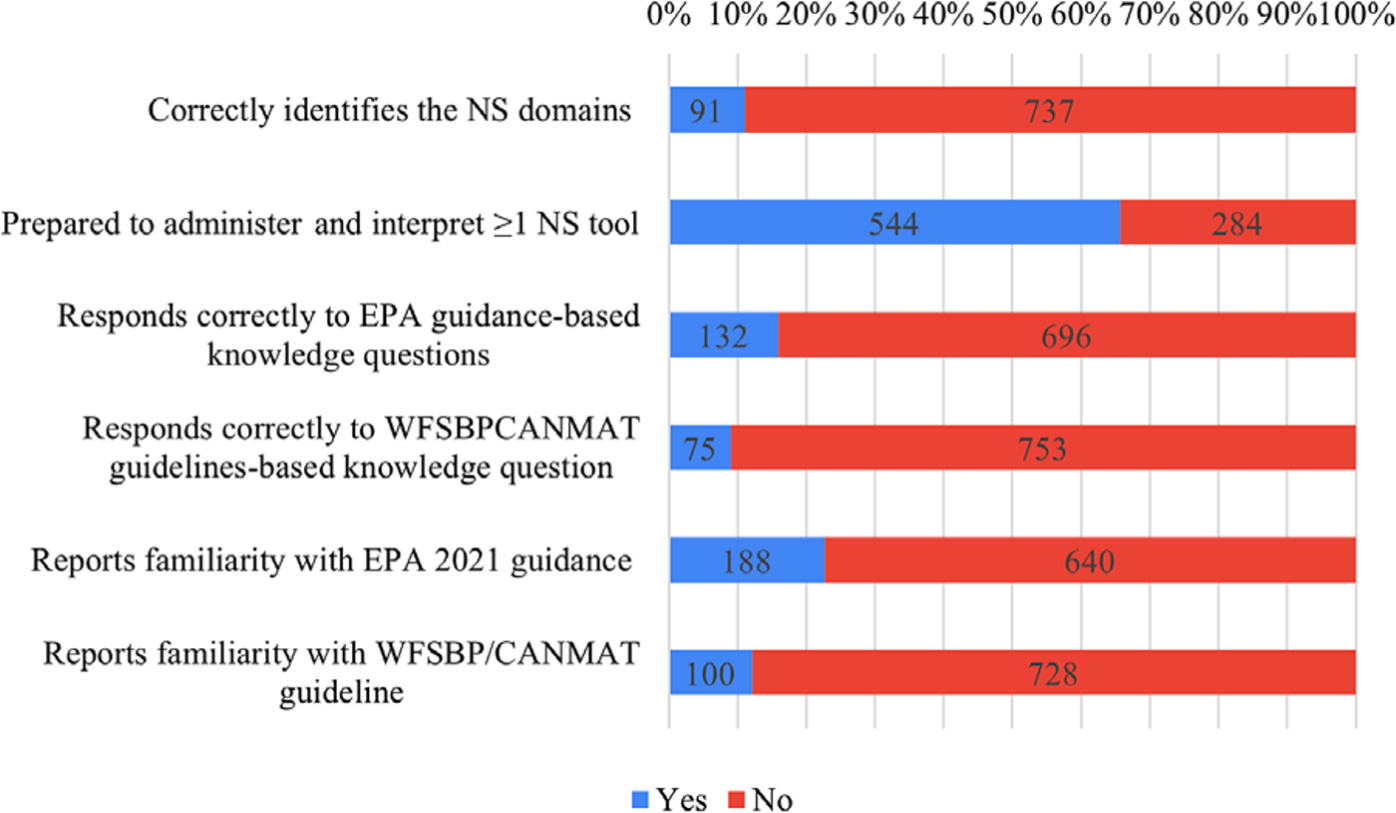
ECPs’ knowledge, skills, and implementation of guidance papers on NS evaluation and management. CANMAT, Canadian Network for Mood and Anxiety Treatments; EPA, European Psychiatric Association; NS, negative symptoms; WFSBP, World Federation of Societies of Biological Psychiatry.

As presented in Figure 191 ECPs (11%) were able to correctly identify NS domains, while 544 (65.7%) reported feeling well-trained to administer and interpret at least 1 scale for NS evaluation. Respectively, 132 (15.9%) and 75 (9.1%) ECPs correctly answered knowledge questions based on the 2021 EPA guidance papers on NS evaluation and management and WFSBP/CANMAT guidelines for the treatment of psychiatric disorders with nutraceuticals and phytoceuticals. Overall, 188 (22.7%) and 100 (12.1%) reported familiarity with the above-mentioned EPA and WFSBP/CANMAT guidance papers (Figure 1).

**Figure 2.**
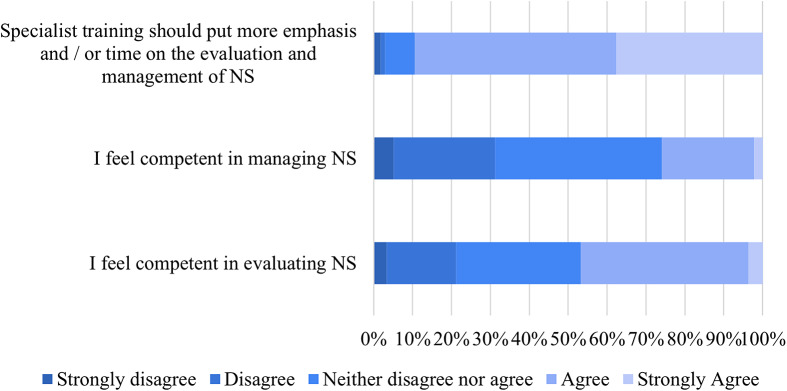
ECPs self-reported sense of competence in NS evaluation and management, and need for more emphasis on the NS in specialist training. NS, negative symptoms.

Regarding the ECPs’ self-reported competence in NS evaluation, respectively, 27 (3.3%) strongly disagreed, 148 (17.9%) disagreed, 266 (32.1%) neither disagreed nor agreed, while 357 (43.1%) agreed, and 30 (3.6%) strongly agreed on feeling competent. As to ECPs’ self-reported competence in NS management, 42 (5.1%) strongly disagreed, 216 (26.1%) disagreed, 356 (43%) neither disagreed nor agreed, while 196 (23.7%) agreed, and 18 (2.2%) strongly agreed in feeling competent. Presented with the statement about the need for putting more emphasis and/or time on NS in the specialist training curriculum, the vast majority of ECPs strongly agreed 311 (37.6%) or agreed 429 (51.8%), while 64 (7.7%) neither disagreed nor agreed, 9 (1.1%) disagreed, and 15 (1.8%) strongly disagreed (Figure 2).

Associations between demographic variables, training characteristics, and knowledge/self-reported sense of competence based on the GLM analyses.

**Figure 3.**
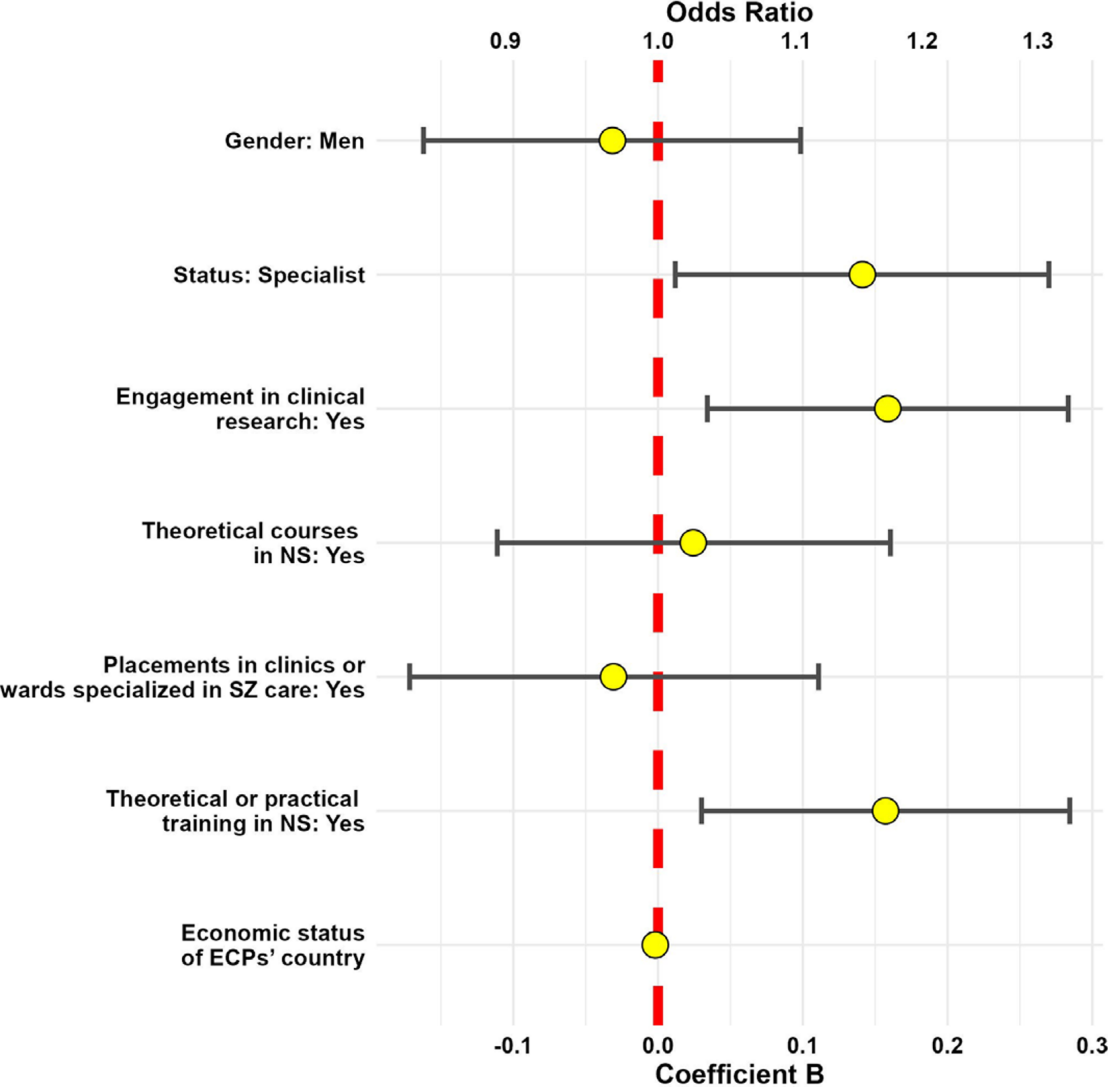
Model 1 predictors of knowledge on NS. The demographics/training characteristics potentially predictive of knowledge on NS are listed on the left side of the figure. The characteristics with an odds ratio greater than 1 (a coefficient beta greater than 0) are statistically significant predictors of knowledge on NS. If the visual representation of the odds ratio/*B* coefficient crosses the red line, it means the tested demographic/training variable is not significantly linked to the knowledge of NS. If the visual representation of the odds ration/B coefficient is localized at the right side of the red line, it means the tested variable is positively linked to the sense of competence in NS evaluation/management. ECPs, early career psychiatrists; NS, negative symptoms, SZ, schizophrenia.

**Figure 4.**
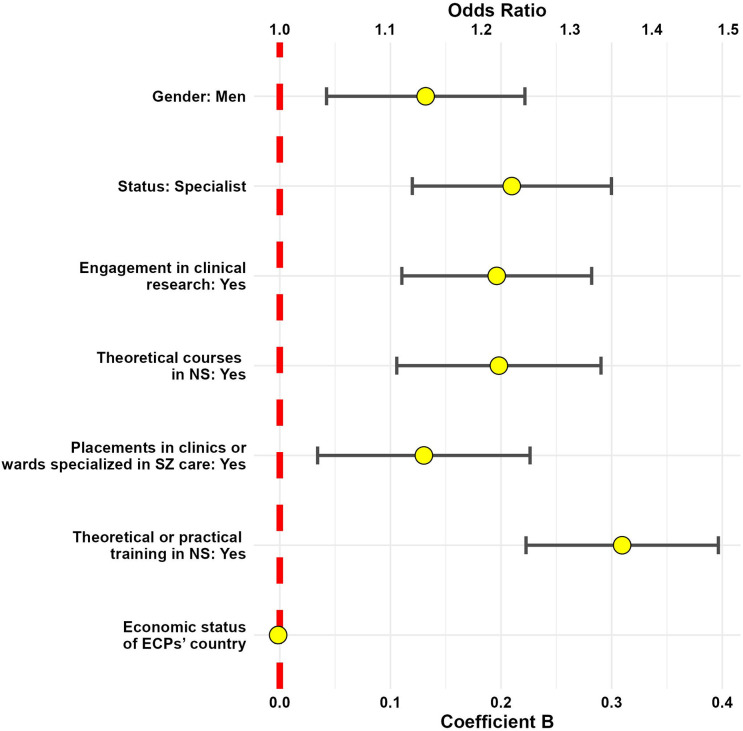
Model 2 predictors of sense of competence. The demographics/training characteristics potentially predictive of sense of competence in NS evaluation and management are listed on the left side of the figure. The characteristics with an odds ratio over 1 (coefficient beta over 0) are statistically significant predictors of sense of competence in NS evaluation and management. If the visual representation of the odds ratio/B coefficient crosses the red line, it means the tested demographic/training variable is not significantly linked to the sense of competence in NS evaluation/ management. If the visual representation of the odds ration/B coefficient is localized at the right side of the red line, it means the tested variable is positively linked to the sense of competence in NS evaluation/management. ECPs, early career psychiatrists; NS, negative symptoms; SZ, schizophrenia.

The first GLM with knowledge as the dependent variable fit the data well, as indicated by the goodness-of-fit statistics (deviance/df = 0.942, Pearson chi-square/df = 0.830, AIC = 2361.694). The Omnibus test was significant (*F* (9, 22) = 22.523, *p* < 0.002), indicating that the model provided a better fit for the data than the intercept-only model. Significant effects were found for: engagement in clinical research (*p* = 0.013), extracurricular training in NS (*p* = 0.015), and specialist status (*p* = 0.032) (Supplementary Table 3, Figure 3). The second model, with sense of competence as the dependent variable, also showed a good fit for the data (Deviance/df = 0.799, Pearson Chi-square/df = 0.770, AIC = 2953.209). The Omnibus test was significant (*χ* ^2^(9) = 186.860, *p* < 0.001). Significant effects were found for: gender (men) (*p* = 0.004), engagement in clinical research (*p* < 0.001), theoretical courses (*p* < 0.001) and placements in clinics/wards specialized in SZ care (*p* < 0.008), extracurricular training (*p* < 0.001), and specialist status (*p* < 0.001) (Supplementary Table 3, Figure 4).

## Discussion

The obtained results indicate that European ECPs, by and large (almost 90%), lack up-to-date knowledge on NS (Figure 2). Only a strikingly low 11% of ECPs correctly identified NS domains, just 15.9% correctly answered questions based on the EPA guidance paper, and merely 9.1% correctly answered the question based on the WFSBP/CANMAT guidelines. These results are at odds with the relatively high proportion of ECPs (65.7%) reporting feeling well-trained to administer and interpret at least one scale to assess NS. One possible reason underlying this contrast could be that ECPs receive training on commonly used but outdated clinical tools (e.g., Positive and Negative Syndrome Scale or Brief Psychiatric Rating Scale), which reflect a different perspective on NS domains, no longer in line with the 2006 consensus statement on NS and with EPA guidance papers [7, 21]. Furthermore, unsatisfactory factual knowledge is a broader issue in the medical field, as previously noted, and actual knowledge is often undervalued in medical training [29]. Interestingly, a study verifying the time progression of knowledge in residents over subsequent years, during specialist training in psychiatry, reported that knowledge did improve in some areas (psychopathology, psychotherapy, somatic therapies), but not in others (development, clinical neuroscience, practice of psychiatry) [30]. This same study mentioned that the program directors managing the training of these residents felt optimistic about their ability to teach the subjects in both areas in which trainees’ knowledge improved and in those it did not [30].

Few ECPs reported being familiar with 2021 EPA guidance on NS evaluation and management (slightly over 22%) and WFSBP/CANMAT guidelines on phytoceuticals and nutraceuticals use (just over 12%), which was reflected in the low rates of correct responses to knowledge questions based on these guidance papers (correspondingly, approximately to 16 and 9%). These observations seem incoherent with the findings that around 70% ECPs report that theoretical courses or placements in clinics/wards specialized in SZ care were part of their specialist training curriculum. This discrepancy may suggest that the training ECPs receive in NS evaluation and management is not grounded in the current knowledge and guidance papers of professional psychiatric organizations. Indeed, GLMs indicated that none of the included specialist training variables were predictive of knowledge on NS. Instead, engagement in clinical research, extracurricular training in NS, and specialist status were associated with updated knowledge on NS. ECPs engaged in clinical research may get more opportunities for training in the use of clinical scales for NS evaluation or participate in conferences that include up-to-date courses in NS evaluation and management (e.g., EPA Congress). On the other hand, ECPs engaged in research could constitute a subgroup of highly motivated individuals with a higher intrinsic drive for knowledge, as it has been shown that almost 90% of European ECPs who engage in research do so (at least partly) after working hours [31]. Specialists are more likely than trainees to be able to cover the costs of the extracurricular training (e.g., conference participation).

A staggering discrepancy was noted between the ECPs’ low levels of knowledge and their higher levels of agreement on feeling competent in NS evaluation (approximately 48%) and management (approximately 25%). Similar dissonance between knowledge and self-perceived confidence in diagnosis and management by mental health professionals was previously reported by Almeida et al. in the field of eating disorders [32]. This might be due to the psychological phenomenon described by Dunning and Kruger in 1999. These authors performed several experiments showing that people who are unskilled in a certain domain tend to overestimate their performance, while at the same time, highly skilled individuals tend to underestimate their abilities [33]. Indeed, this effect was previously reported in medical students and physicians of various specialties [34–36]. Our work mirrors previous observations showing that women rate their performance lower than men [37], even despite presenting a similar level of competence [38]. Prior reports also noted that more time spent on learning and practicing a skill enhances the self-reported performance regardless of the objective improvement [34]. The CARE study results align with these literature observations, indicating that theoretical training/placements in clinics/wards specializing in SZ care, as part of the training curriculum, are predictive of higher self-rated competence in NS evaluation and management, despite not being predictive of knowledge on NS.

The lack of association between theoretical courses in NS/placements in specialized SZ care clinics/wards and knowledge on NS, together with the overwhelming (almost 90%) ECPs’ expression of need for more training in NS, should be considered by institutions responsible for specialist training curricula in the European countries, as well as professional organizations striving for best practice in mental health care. One possible strategy to bridge this gap is to implement structured workshops with real-life simulations and promote clinical rotations focused on assessing and managing NS, which would be developed on the basis of EPA guidelines on NS assessment and management. The need for more training, as expressed by ECPs, is in line with the recent call to optimize education for mental health professionals to improve outcomes for people with SZ [2]. As Schuwirth and van der Vleuten noted [39], students learn what they know will later be inspected. Therefore, we suggest that EPA guidelines on NS assessment and management should be integrated into mandatory specialist training curricula as well as national specialist exams. Moreover, the European Board Exam of Psychiatry, which sets out to impact the learning outcomes for psychiatrists and balance learning standards [40], could play a significant role in redirecting the ECPs’ attention to the most critical themes in psychiatry, including NS of SZ.

## Limitations

The limitations of this work pertain to its methodology, specifically the use of a limited, convenience sample; therefore, the obtained results may not be representative of the general ECP population in Europe. A precise number of ECPs in Europe is very hard to gauge based on the available data. A survey in 2015 estimated that there are some 46 thousands of ECPs in Europe [41]; however, these data need to be updated as more recent data suggest that the number of psychiatrists has decreased among European region countries [42]. On the other hand, it is also hard to discern how big a part of the real-life ECP population is constituted by the assessed sample, as this study only examined 19 of the 44 European countries. These limitations have been discussed more broadly in the study protocol [24]. Our sample of ECPs might not be representative of all the European ECPs, but it represents a sample of ECPs who are responsive, as they are involved in some research activities. Notably, the child and adolescent ECPs constituted only 10% of the studied group, which is likely because their numbers per capita in European countries are even lower than those for adult psychiatrists. Although the convenience sampling approach has inherent limitations, structured dissemination through EPA-affiliated societies and EFPT networks ensured broad and diverse participation from ECPs across Europe. Moreover, the assessed sample is comparable to that of studies conducted previously with a similar methodology [31, 43–45], and the obtained results should prompt action in refining psychiatric training, even if they are reflective only of some European ECPs.

## Conclusions

The CARE study results revealed significant gaps in European ECPs’ knowledge on NS, coupled with a widely expressed demand for more emphasis and/or time dedicated to NS in specialist training curricula. These data should be considered by institutions responsible for specialist training in psychiatry in Europe, with a focus on providing up-to-date knowledge and skills for ECPs. Providing ECPs with state-of-the-art training in knowledge and skills in the evaluation and management of NS is essential to promote the best outcomes for people with SZ. Addressing these gaps could transform SZ care by improving the recognition of NS among SZ patients and increasing the implementation of recommended treatment strategies, which should ultimately result in improved functional outcomes and better quality of life for people with SZ. Further longitudinal studies to track eventual post-intervention improvements are much needed to verify the effects of recommended therapies in real-life samples of patients with SZ.

## Supporting information

10.1192/j.eurpsy.2025.10142.sm001 Krupa et al. supplementary material Krupa et al. supplementary material

## Supplementary material

The supplementary material for this article can be found at http://doi.org/10.1192/j.eurpsy.2025.10142.
