# Supplementary material for: Early career psychiatrists’ ability to evaluate and manage negative symptoms of schizophrenia – Results from a European survey
Source: Eur Psychiatry. 2025 Dec 17;69(1):e11. doi: 10.1192/j.eurpsy.2025.10142 (PMC12816925; doi:10.1192/j.eurpsy.2025.10142)
Supplement: Krupa et al. supplementary material [file S0924933825101429sup001.docx]

Supplementary Table 1. General group characteristics and comparisons between included vs. excluded observations

| Demographic and training/work experience characteristics | Observations included in the analysis  n= 828 | Observations excluded from the analysis  n= 41 | Comparison between included and excluded observations |
| --- | --- | --- | --- |
| Gender (Female) n (%) | 523 (63.2%) | 23 (56.1%) | p= 0.462^a^ |
| Number of years of experience in the mental health system [median (IQR)] | 4 (2 – 6) | 6 (5 – 8.5) | **p< 0.001^b^** |
| Trainee status (vs. specialist) in adult or child and adolescent psychiatry n (%) | 545 (65.8%) | 13 (31.7%) | **p< 0.001^a^** |
| Confirms engagement in clinical research n (%) | 382 (46.1%) | 17 (41.5%) | p= 0.558^a^ |
| Confirms theoretical courses on the NS of SZ were included in the specialist training curriculum n (%) | 562 (67.9%) | 28 (68.3%) | p= 0.955^a^ |
| Confirms placements in clinics / wards specialized in SZ care and management were included in the specialist training curriculum n (%) | 582 (70.3%) | 29 (70.7%) | p= 0.952^a^ |
| Participated in additional theoretical or practical training in the NS of SZ assessment and management (outside of specialist training programme) n (%) | 423 (51.1%) | 21 (51.2%) | p= 0.987^a^ |
| Country n (%) | Bosnia and Herzegovina 41 (5%)  Bulgaria 19 (2.3%)  Croatia 28 (3.4%)  Denmark 39 (4.7%)  France 35 (4.2%)  Germany 40 (4.8%)  Hungary 23 (2.8%)  Italy 114 (13.8%)  Latvia 19 (2.3%)  Lithuania 16 (1.9%)  North Macedonia 17 (2.1%)  Poland 147 (17.8%)  Portugal 15 (1.8%)  Romania 61 (7.4%)  Serbia 16 (1.9%)  Spain 56 (6.8%)  Switzerland 49 (5.9%)  Turkey 64 (7.7%)  United Kingdom of Great Britain and Northern Ireland 29 (3.5%) | Albania 2 (4.9%)  Armenia 1 (2.4%)  Austria 1 (2.4%)  Belgium 1 (2.4%)  Cyprus 1 (2.4%)  Czech Republic 12 (29.3%)  Greece 4 (9.8%)  Malta 1 (2.4%)  Netherlands 2 (4.9%)  Norway 1 (2.4%)  Slovakia 3 (7.3%)  Slovenia 1 (2.4%)  Sweden 8 (19.5%)  Ukraine 3 (7.3%) | - |
| GDP per capita in thousands of US dollars [median (IQR)] | 26.8 (21.42 – 41.09) | 33.04 (26.44 – 58.1) | p= 0.092^b^ |

^a^-ꭓ2- test, ^b^- Mann-Whitney U test, GDP- gross domestic product, IQR- interquartile range, NS- negative symptoms, SZ- schizophrenia, US- United States of America

Supplementary Table 2. Knowledge, skills, self-reported sense of confidence in evaluating and managing NS and implementation of NS guidance with comparisons between included vs. excluded observations

| Variables | Observations included in the analysis | Observations excluded from the analysis | Comparison between included and excluded observations |
| --- | --- | --- | --- |
| Correctly identifies the NS domains n (%) | 91 (11%) | 4 (9.8%) | p> 0.99^a^ |
| Reports feeling well-trained to administer and interpret at least one tool for the assessment of the NS n (%) | 544 (65.7%) | 28 (63.8%) | p= 0.733^a^ |
| Correctly responds to 1^st^ EPA knowledge question on pharmacological management n (%) | 611 (73.8%) | 31 (75.6%) | p= 0.796^a^ |
| Correctly responds to EPA knowledge question on non-pharmacological management n (%) | 350 (42.3%) | 18 (43.9%) | p= 0.836^a^ |
| Correctly responds to 2^nd^ EPA knowledge question on pharmacological management n (%) | 360 (43.5%) | 20 (48.8%) | p= 0.504^a^ |
| Total correct responses responds to all EPA knowledge questions n (%) (0/1/2/3) | 83 (10%)  301 (36.4%)  312 (37.7%)  132 (15.9%) | 1 (2.4%)  14 (34.1%)  23 (56.1%)  3 (7.3%) | p= 0.053^a^ |
| Correctly responds to WFSBP/CANMAT knowledge question n (%) | 75 (9.1%) | 6 (14.6%) | p= 0.263^a^ |
| Total correct responses to ALL knowledge questions n (%) (0/1/2/3/4) | 76 (9.2%)  285 (34.4%)  297(35.9% )  163 (19.7%)  7 (0.8%) | 1 (2.4%)  11 (26.8%)  23 (56.1%)  6 (14.6%)  0 (0%) | p= 0.097^a^ |
| Self-reports sense of competence in evaluation of the NS n (%) (strongly disagree/disagree/neither disagree nor agree/agree/strongly agree) | 27 (3.3%)  148 (17.9%)  266 (32.1%)  357 (43.1%)  30 (3.6%) | 1 (2.4%)  4 (9.8%)  12 (29.3%)  22 (53.7%)  2 (4.9%) | p= 0.589^a^ |
| Self-reports sense of competence in management of the NS n (%) (strongly disagree/disagree/neither disagree nor agree/agree/strongly agree) | 42 (5.1%)  **216 (26.1%)**  356 (43%)  **196 (23.7%)**  18 (2.2%) | 1 (2.4%)  **4 (9.8%)**  16 (39%)  **18 (43.9%)**  2 (4.9%) | **p= 0.014^a^** |
| Reports familiarity with EPA 2021 guidance n (%) | 188 (22.7%) | 10 (24.4%) | p= 0.802^a^ |
| Reports familiarity with WFSBP/CANMAT guidelines n (%) | 100 (12.1%) | 4 (9.8%) | p= 0.808^a^ |
| Agrees that there should be more emphasis on the NS in specialist training n (%) (strongly disagree/disagree/neither disagree nor agree/agree/strongly agree) | 15 (1.8%)  9 (1.1%)  64 (7.7%)  429 (51.8%)  311 (37.6%) | 2 (4.9%)  0 (0.0%)  4 (9.8%)  24 (58.5%)  11 (26.8%) | p= 0.395^a^ |

^a^-ꭓ2- test, NS- negative symptoms, PS- positive symptoms, columns with responses for which significantly different proportion of observations were written in bold

Supplementary Table 3. GLMs exploring the variables associated with knowledge and sense of competence.

| **Model 1 Predictors of knowledge on NS** | | | | | | | | | | |
| --- | --- | --- | --- | --- | --- | --- | --- | --- | --- | --- |
| Parameter | B | Std. Error | 95% Wald CI | | Hypothesis Test | | | Exp(B) | 95% Wald CI for Exp(B) | |
|  |  |  | Lower | Upper | Wald Chi-Square | df | Sig. |  | Lower | Upper |
| **Intercept** | -0.970 | 0.0892 | -1.145 | -0.795 | 118.293 | 1 | **<0.001** | **0.379** | **0.318** | **0.451** |
| Gender: Male | -0.031 | 0.0665 | -0.162 | 0.099 | 0.224 | 1 | 0.636 | 0.969 | 0.851 | 1.104 |
| **Specialist status** | 0.141 | 0.0658 | 0.012 | 0.270 | 4.598 | 1 | **0.032** | **1.152** | **1.012** | **1.310** |
| **Engagement in clinical research** | 0.159 | 0.0636 | 0.034 | 0.283 | 6.227 | 1 | **0.013** | **1.172** | **1.035** | **1.328** |
| Theoretical courses on NS | 0.024 | 0.0693 | -0.111 | 0.160 | 0.123 | 1 | 0.726 | 1.025 | 0.895 | 1.174 |
| Placements in clinics / wards specialized in SZ care | -0.031 | 0.0721 | -0.172 | 0.110 | 0.182 | 1 | 0.669 | 0.970 | 0.842 | 1.117 |
| **Extracurricular training in NS** | 0.157 | 0.0649 | 0.030 | 0.284 | 5.866 | 1 | **0.015** | **1.170** | **1.030** | **1.329** |
| Economic status of the ECPs’ country | -0.002 | 0.0014 | -0.005 | 0.001 | 1.817 | 1 | 0.178 | 0.998 | 0.995 | 1.001 |

| **Model 2 Predictors of sense of competence** | | | | | | | | | | |
| --- | --- | --- | --- | --- | --- | --- | --- | --- | --- | --- |
| Parameter | B | Std. Error | 95% Wald CI | | Hypothesis Test | | | Exp(B) | 95% Wald CI for Exp(B) | |
|  |  |  | Lower | Upper | Wald Chi-Square | df | Sig. |  | Lower | Upper |
| **Intercept** | -0.286 | 0.0601 | -0.404 | -0.168 | 22.684 | 1 | **<0.001** | **0.751** | **0.668** | **0.845** |
| **Gender: Male** | 0.132 | 0.0457 | 0.042 | 0.222 | 8.313 | 1 | 0.004 | 1.141 | 1.043 | 1.248 |
| **Specialist status** | 0.210 | 0.0459 | 0.120 | 0.300 | 20.855 | 1 | **0.000** | **1.233** | **1.127** | **1.349** |
| **Engagement in clinical research** | 0.196 | 0.0438 | 0.110 | 0.282 | 20.076 | 1 | **0.000** | **1.217** | **1.117** | **1.326** |
| **Theoretical courses on NS** | 0.198 | 0.0471 | 0.106 | 0.290 | 17.692 | 1 | **0.000** | 1.219 | 1.112 | 1.337 |
| **Placements in clinics / wards specialized in SZ care** | 0.130 | 0.0490 | 0.034 | 0.226 | 7.071 | 1 | **0.008** | **1.139** | **1.035** | **1.254** |
| **Extracurricular training in NS** | 0.309 | 0.0444 | 0.223 | 0.396 | 48.687 | 1 | **0.000** | **1.363** | **1.249** | **1.486** |
| Economic status of the ECPs’ country | -0.001 | 0.0010 | -0.003 | 0.000 | 2.362 | 1 | 0.124 | 0.999 | 0.997 | 1.000 |

B- unstandardized GLM coefficient. CI- confidence interval. Exp(B)- exponential of the GLM coefficient (odds ratio), GDP- gross income per capita, NS- negative symptoms, Sig.- significance
